# Lifespan Extension and Increased Resistance to Environmental Stressors Induced by 5-Hydroxy-4-phenylfuran-2(5H)-one in *Caenorhabditis elegans*

**DOI:** 10.3390/ijms27209208

**Published:** 2026-10-16

**Authors:** Yasunori Okuma, Kayo Yasuda, Sumino Yanase, Naoaki Ishii, Yoshio Tsujino

**Affiliations:** 1 Research Centre, Arimino Co., Ltd., 25-5 Miyako, Namegawa-machi, Hiki-gun 355-0812, Saitama, Japan; 215p402p@gsuite.kobe-u.ac.jp; 2 Graduate School of Science, Technology and Innovation, Kobe University, Kobe-shi 657-8501, Hyogo, Japan; 3 Department of Health Management, School of Health Studies, Tokai University, Hiratsuka-shi 259-1292, Kanagawa, Japan; 4 Department of Health Science, School of Sports & Health Science, Daito Bunka University, Higashimatsuyama-shi 355-8501, Saitama, Japan

**Keywords:** *Caenorhabditis elegans*, ageing, antioxidant, lifespan, longevity

## Abstract

This study investigated whether 5-hydroxy-4-phenylfuran-2(5H)-one (FG1), a bioactive compound derived from aromatic vinegar, exerts anti-ageing effects in the nematode *Caenorhabditis elegans*. FG1 treatment significantly extended both mean and maximum lifespan and preserved locomotor activity during ageing. Transcriptomic analysis revealed that FG1 upregulated genes associated with detoxification, glutathione metabolism, oxidative phosphorylation, and longevity-regulating pathways. Gene ontology and pathway analyses suggested activation of antioxidant and xenobiotic defence systems. FG1 significantly extended lifespan in wild-type worms, whereas no statistically significant lifespan extension was detected in SKN-1-deficient worms, suggesting a possible involvement of SKN-1 signalling in the lifespan-extending effect of FG1. FG1 also reduced lipofuscin and hydrogen peroxide accumulation in vivo and enhanced resistance to heat- and paraquat-induced oxidative stress. Overall, these findings suggest that the effects of FG1 on longevity and stress resistance are associated with changes in antioxidant and detoxification responses, although the contribution of SKN-1 requires further investigation.

## 1. Introduction

Global population ageing is accelerating at an unprecedented rate and is emerging as one of the most significant demographic trends of the 21st century. Ageing has profound implications not only for economies and healthcare systems but also for the overall structure and sustainability of societies, thereby making the promotion of healthy ageing a major global challenge [1]. According to the World Health Organization, by 2030, one in six individuals worldwide will be aged ≥ 60 years. The number of people aged ≥ 60 years is expected to increase from 1 billion in 2020 to 1.4 billion by 2030, and double again to 2.1 billion by 2050. Moreover, the population aged ≥ 80 years is projected to triple between 2020 and 2050, reaching approximately 426 million individuals [2]. This unprecedented demographic shift underscores the urgent need to elucidate the biological mechanisms of ageing and to develop novel interventions that can extend healthspan—the period of life spent in good health—rather than just lifespan.

Ageing is not solely a chronological process but rather a multifactorial biological phenomenon that involves the progressive accumulation of molecular and cellular damage over time. These alterations ultimately lead to functional decline and increased vulnerability to disease. The hallmarks of ageing include genomic instability, telomere attrition, epigenetic alterations, loss of proteostasis, impaired macroautophagy, dysregulated nutrient sensing, mitochondrial dysfunction, cellular senescence, stem cell exhaustion, altered intercellular communication, chronic inflammation, and dysbiosis [3]. These hallmarks are not independent; rather, they interact in complex feedback loops that amplify the physiological deterioration associated with ageing.

Although the intrinsic biological mechanisms of ageing are fundamental, extrinsic factors, such as nutrition, lifestyle, psychological stress, and exposure to environmental toxins, also play critical roles in determining the rate and quality of ageing. Dietary and natural bioactive compounds modulate several ageing-related pathways, offering potential strategies to promote longevity and mitigate age-associated diseases. Thus, dietary interventions and nutraceutical approaches represent promising, non-invasive avenues for promoting healthy ageing at the population level.

Fraglide-1 (5-hydroxy-4-phenylfuran-2(5H)-one, FG1), a bioactive lactone, is a compelling example of a potent bioactive compound derived from traditional foods, offering significant therapeutic potential. FG1, a compound isolated from Kozu (Chinese Zhenjiang aromatic vinegar), has garnered growing scientific interest due to its diverse biological activities [4]. FG1 activates cellular defence systems against oxidative damage, inhibits NF-κB-mediated pro-inflammatory signalling, and modulates energy metabolism, thereby influencing key biological pathways implicated in cellular homeostasis. Because oxidative stress, inflammation, and metabolic imbalance are central hallmarks of ageing [5,6,7], these pleiotropic actions of FG1 suggest that it may exert a geroprotective or longevity-promoting effect.

The nematode *Caenorhabditis elegans* serves as a powerful model organism for ageing research because of its short lifespan, well-characterised genetics, and conserved molecular pathways that are homologous to those in humans. Approximately two-thirds of human disease-associated genes have identifiable *C. elegans* counterparts [8]. Importantly, *C. elegans* enables assessment of the systemic effects of bioactive compounds on whole-organism physiology, encompassing neural, muscular, and metabolic networks. Over the past decades, it has become a key model system for elucidating the mechanisms of longevity and for screening natural products with anti-ageing potential.

Given these considerations, the present study aimed to evaluate whether FG1 extends the lifespan of *C. elegans*. Furthermore, we sought to elucidate the molecular mechanisms underlying FG1-mediated lifespan extension by focusing on its potential roles in oxidative stress resistance, metabolic regulation, and longevity-associated cellular signalling pathways.

## 2. Results

### 2.1. Effects of FG1 on the Lifespan and Locomotive Capability of C. elegans

**Figure 1:**
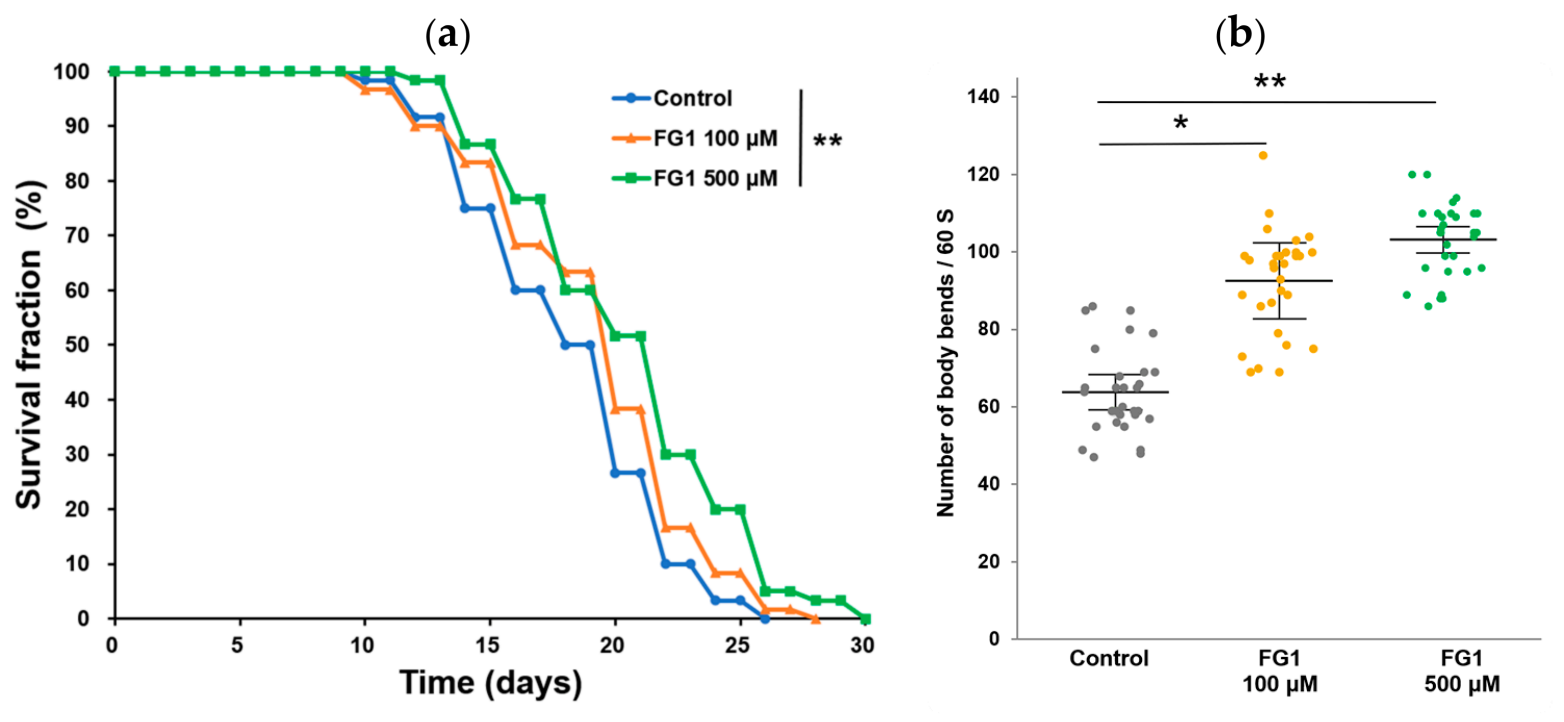
(**a**) Effects of FG1 on the lifespan of *C. elegans*. Five-day-old DH26 (*rrf-3(b26)*) mutants were transferred to NGM plates containing 0, 100, or 500 μM FG1 and maintained at 25 °C (final DMSO concentration: 0.1% in all groups). Survival was monitored every 2 days based on touch response until death. Survival curves represent three independent trials (*n* > 65 worms/group). Survival differences were tested for significance using the log-rank test. Asterisks indicate significant differences compared to the control at ** *p* < 0.01. (**b**) Effects of FG1 on locomotor capability. Five-day-old N2 worms were treated with varying FG1 concentrations on NGM plates containing 0, 100, or 500 μM FG1 at 20 °C until day 10 (final DMSO concentration: 0.1% in all groups). Bending rates were measured in S-basal buffer for 1 min following a 2-min recovery period. Lines in the scatterplot represent mean ± SD (*n* = 10 worms/trial, 3 independent trials). Asterisks indicate significant differences compared to each control treatment at * *p* < 0.05 and ** *p* < 0.01.

First, the effects of FG1 (0, 100, and 500 µM) on the lifespan of *C. elegans* were examined. As shown in Figure 1a and summarized in Table 1, the mean lifespan of worms treated with 500 µM FG1 was significantly increased compared with that of the control group. The mean and maximum lifespans of worms treated with 500 µM of FG1 were extended by 11.9% and 26.4%, respectively. This suggests that FG1 may help slow ageing in *C. elegans*.

Locomotion rate is also considered to be closely related to longevity [9]. Therefore, to investigate whether FG1 improved locomotor healthspan during ageing, we measured body bending rates. As shown in Figure 1b, the number of body bends was significantly higher in the FG1-treated group. These results suggest that FG1 contributes to the maintenance of physiological function during ageing by preserving locomotor activity.

### 2.2. Effects of FG1 Treatment on C. elegans Transcriptome Differential Gene Expression

To investigate the effects of FG1 treatment on *C. elegans*, RNA sequencing data were analysed to identify differentially expressed genes (DEGs) between the treatment groups, using false discovery rate (FDR)-adjusted *p*-value < 0.05 and |fold change| ≥ 1.5 as cut-off criteria.

**Figure 2:**
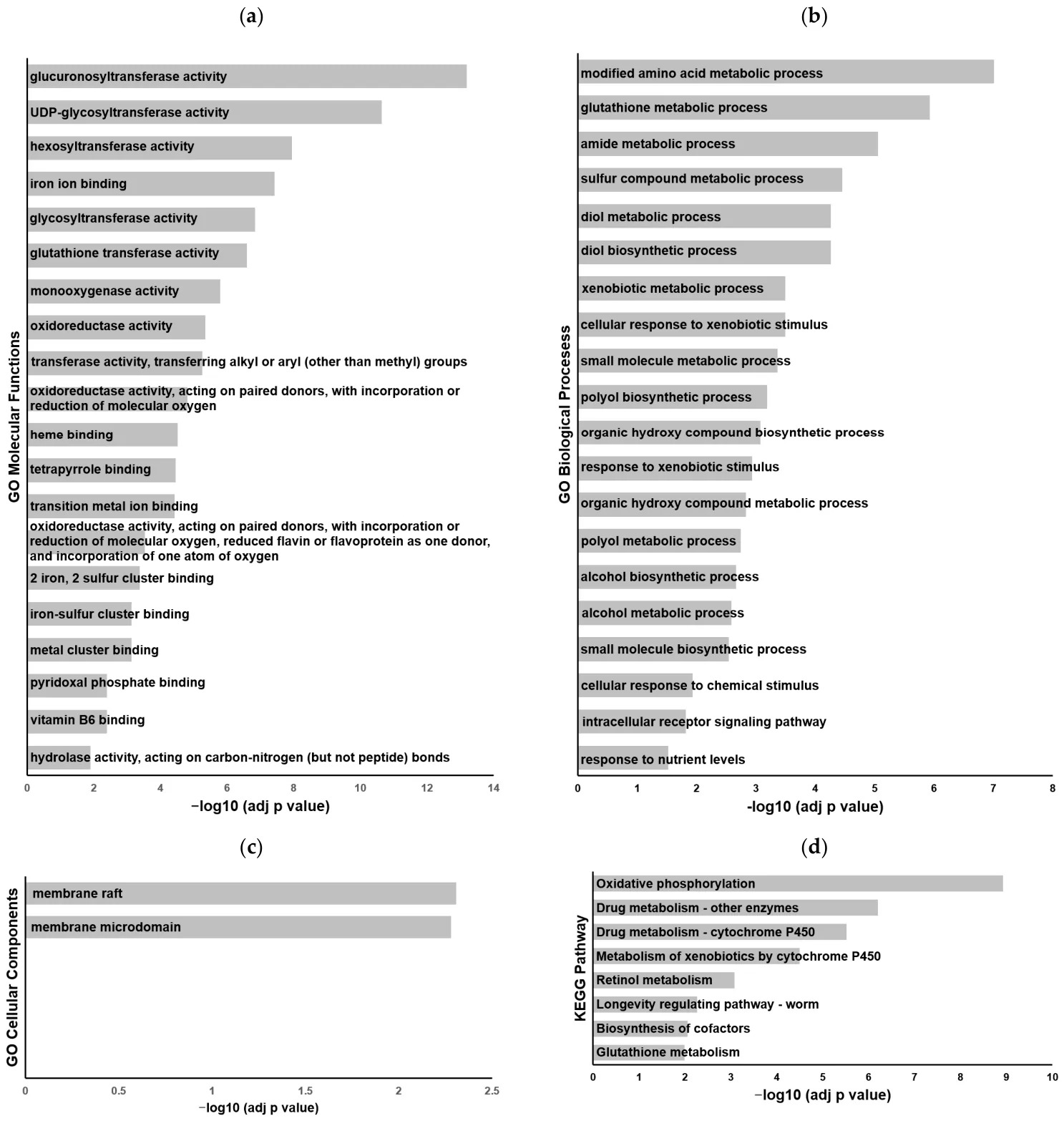
Gene ontology (GO) and Kyoto Encyclopedia of Genes and Genomes (KEGG) analysis plots of differentially expressed genes (DEGs) across treatments. (**a**) GO molecular function analysis; (**b**) GO biological process analysis; (**c**) GO cellular component analysis; (**d**) KEGG pathway analysis. Approximately 2000 5-day-old DH26 worms were transferred to NGM plates containing 0 or 500 μM FG1 and collected at day 6. Worms were washed three times with S-basal buffer, and three independent biological replicates (*n* = 3 per group) were prepared for RNA-seq, with each replicate consisting of approximately 2000 pooled worms.

GO pathway enrichment analysis was performed based on the DEGs of FG1-treated worms using threshold-corrected *p*-values < 0.05. A total of 240 GO terms were significantly enriched based on the DEGs. Molecular functions primarily included glucuronosyltransferase, UDP-glycosyltransferase, hexosyltransferase, iron ion binding, glycosyltransferase, and glutathione transferase activities. Genes encoding proteins with oxidoreductase activity and haeme-binding activity were also induced. Biological processes mainly included modified amino acid, glutathione, amide, sulphur compound, and diol metabolism. Cellular components primarily included membrane rafts and microdomains (Figure 2a–c). KEGG-based pathway analysis using DEG data revealed that nematodes exposed to FG1 modulated the oxidative phosphorylation, drug metabolism, xenobiotic metabolism by cytochrome P450, retinol metabolism, and longevity-regulating pathways (Figure 2d).

Based on these observations, we classified the gene expression changes among the gene groups into two types. Type I changes are closely linked to the detoxification genes. Xenobiotic detoxification occurs in three phases: phase I (in which cytochrome P450 enzymes (CYPs) chemically modify toxicants); phase II (in which UDP-glucuronosyl transferases and glutathione S-transferases (GSTs) increase their solubility); and phase III (in which modified endotoxins are released into the extracellular space by ATP-binding cassette transporters). Type II changes are closely linked to mitochondria. These organelles supply energy to organisms through oxidative phosphorylation, in which iron ions modulate the entire mitochondrial energy metabolic process.

**Figure 3:**
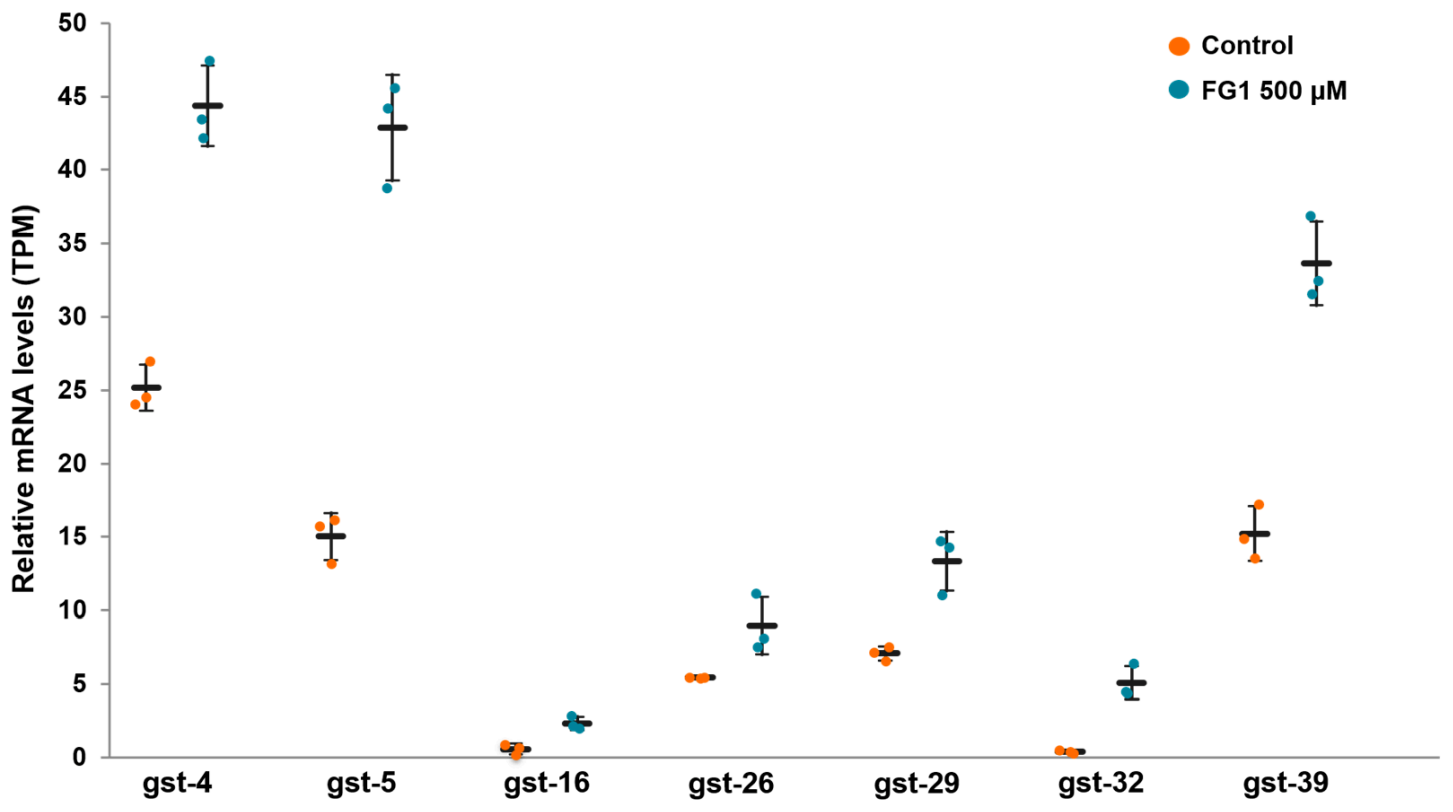
Gene-level analysis of selected GST family members identified in the RNA-seq dataset. Individual expression levels of GST family genes that met the predefined differential-expression criteria in the RNA-seq analysis (FDR-adjusted *p*-value < 0.05 and |fold change| ≥ 1.5). Data are presented as individual data points with mean ± SD from three independent biological replicates.

To further characterize the transcriptomic changes identified in the RNA-seq dataset, we examined the expression patterns of individual GST family members that met the predefined differential-expression criteria for differential expression in the RNA-seq analysis (false discovery rate (FDR)-adjusted *p*-value < 0.05 and |fold change| ≥ 1.5). This targeted analysis showed increased expression of multiple *gst* genes, including *gst-4*, *gst-5*, *gst-16*, *gst-26*, *gst-29*, *gst-32*, and *gst-39* (Figure 3). These results provide additional detail on the individual *gst* genes contributing to the enrichment of glutathione- and detoxification-related responses observed in the transcriptomic analysis.

### 2.3. Effects of FG1 on the Redox-Active Signalling Pathway

**Figure 4:**
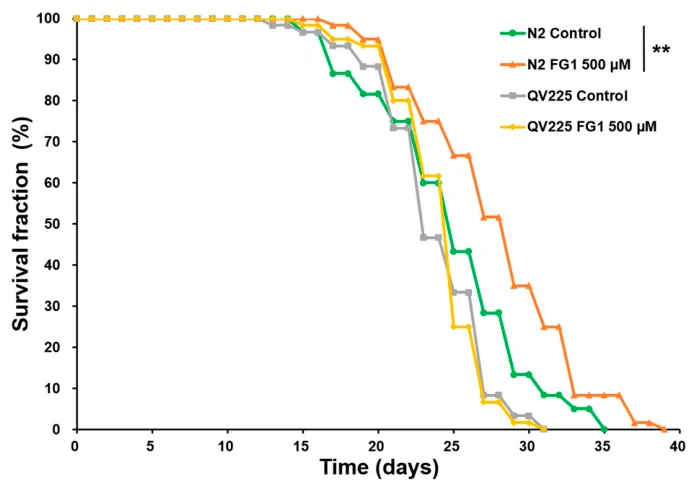
Effects of FG1 on the lifespan of *C. elegans* N2 and QV225 strains. Five-day-old N2 wild-type and QV225 mutants were transferred to NGM plates containing 0 or 500 μM FG1 and maintained at 20 °C (final DMSO concentration: 0.1% in all groups). Survival was monitored every 2 days based on touch response until death. Survival curves represent three independent trials (*n* > 65 worms/group). Survival differences were tested for significance using the log-rank test. Asterisks indicate significant differences compared to the respective control treatment within each genotype (** *p* < 0.01).

RNA-seq revealed that FG1 treatment modulated the expression of genes associated with UDP-glycosyltransferase, glycosyltransferase, and glutathione transferase activities, suggesting the involvement of a redox-active signalling pathway. In *C. elegans*, this pathway is regulated by SKN-1, an orthologue of mammalian Nrf2 [10]. To assess the role of SKN-1, lifespan assays were conducted using the SKN-1-deficient strain QV225 (*skn-1(zj15)*). In statistical comparisons performed within each genotype, while FG1 (500 µM) significantly extended the lifespan of N2 wild-type worms (*p* < 0.01), this lifespan-extending effect was not observed in the SKN-1-deficient strain QV225, with no statistically significant difference observed between the control and FG1-treated groups (*p* > 0.05, log-rank test; Figure 4 and summarized in Table 2). These findings are consistent with a possible involvement of SKN-1 signalling in the lifespan-extending effect of FG1. However, because the statistical comparisons were performed separately within each genotype, these results alone do not establish that the response to FG1 differs significantly between genotypes.

### 2.4. Effects of FG1 on In Vivo Antioxidant Capacity in C. elegans

To determine whether FG1 mitigates endogenous oxidative damage during natural ageing, we measured lipofuscin accumulation and intracellular H_2_O_2_ levels in middle-aged worms under unperturbed physiological conditions.

**Figure 5:**
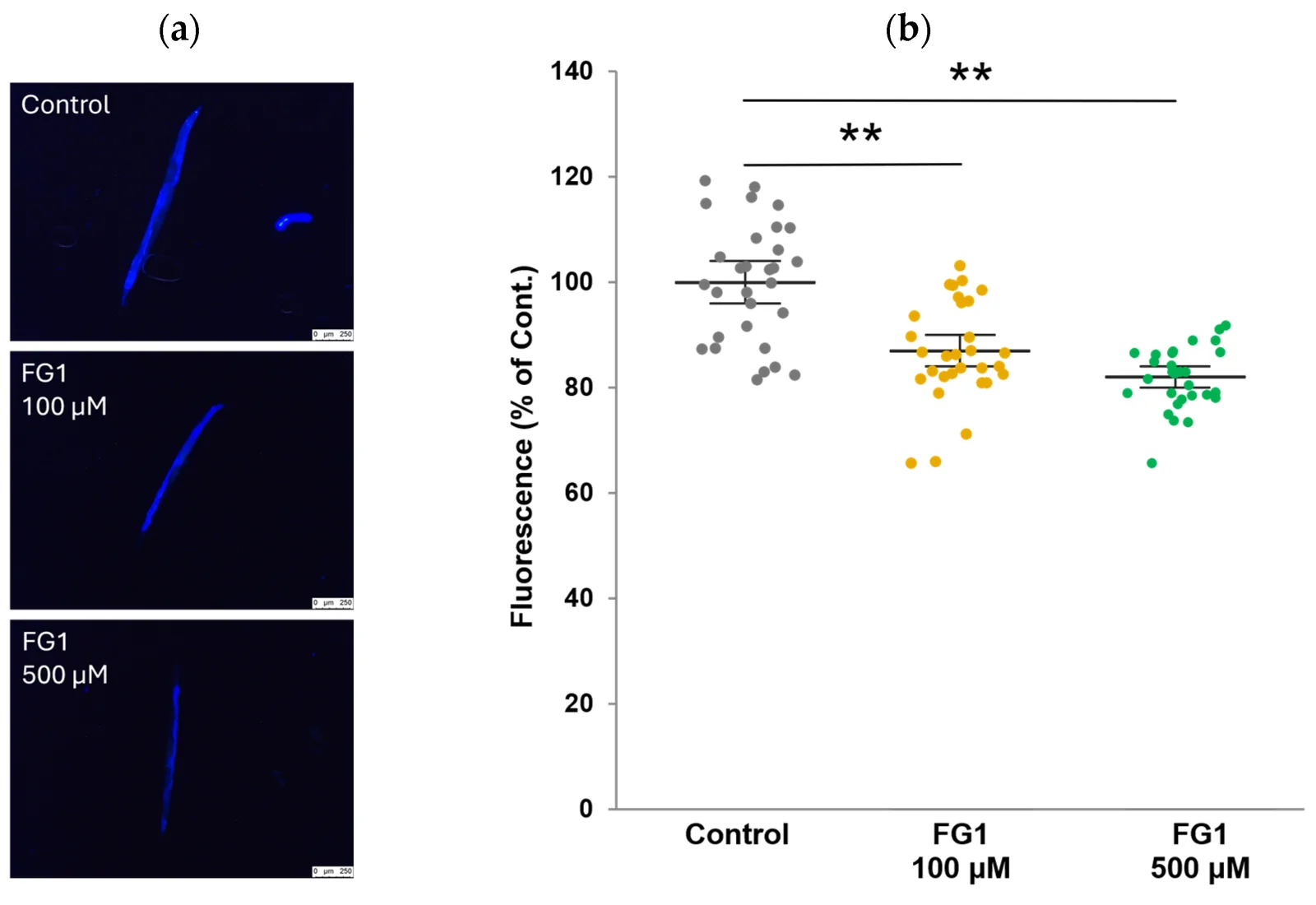
Effects of FG1 on lipofuscin accumulation. Five-day-old N2 worms were cultured on NGM plates with FG1 0, 100 or 500 μM for 10 days (final DMSO concentration: 0.1% in all groups). At 15 days of age, lipofuscin was measured by assessing autofluorescence. (**a**) Representative images of worms are shown. (**b**) Autofluorescence of lipofuscin was quantified using ImageJ software (version 1.54p). Each dot represents an individual worm, and different colours indicate the different FG1 treatment groups. Lines in the scatterplot represent mean ± SD (*n* = 10 worms/trial, 3 independent trials). Asterisks indicate significant differences compared to each control treatment at ** *p* < 0.01.

Lipofuscin accumulation is an established marker of ageing in *C. elegans* [11]. Lipofuscin, a lipid peroxidation by-product, is assessed via autofluorescence and increases slowly during adulthood in the reproductive phase and more rapidly after reproduction. Therefore, its accumulation reflects physiological rather than chronological ageing. In this study, autofluorescence intensity was significantly reduced in worms treated with 100 µM and 500 µM FG1 compared to 0 µM controls (Figure 5).

**Figure 6:**
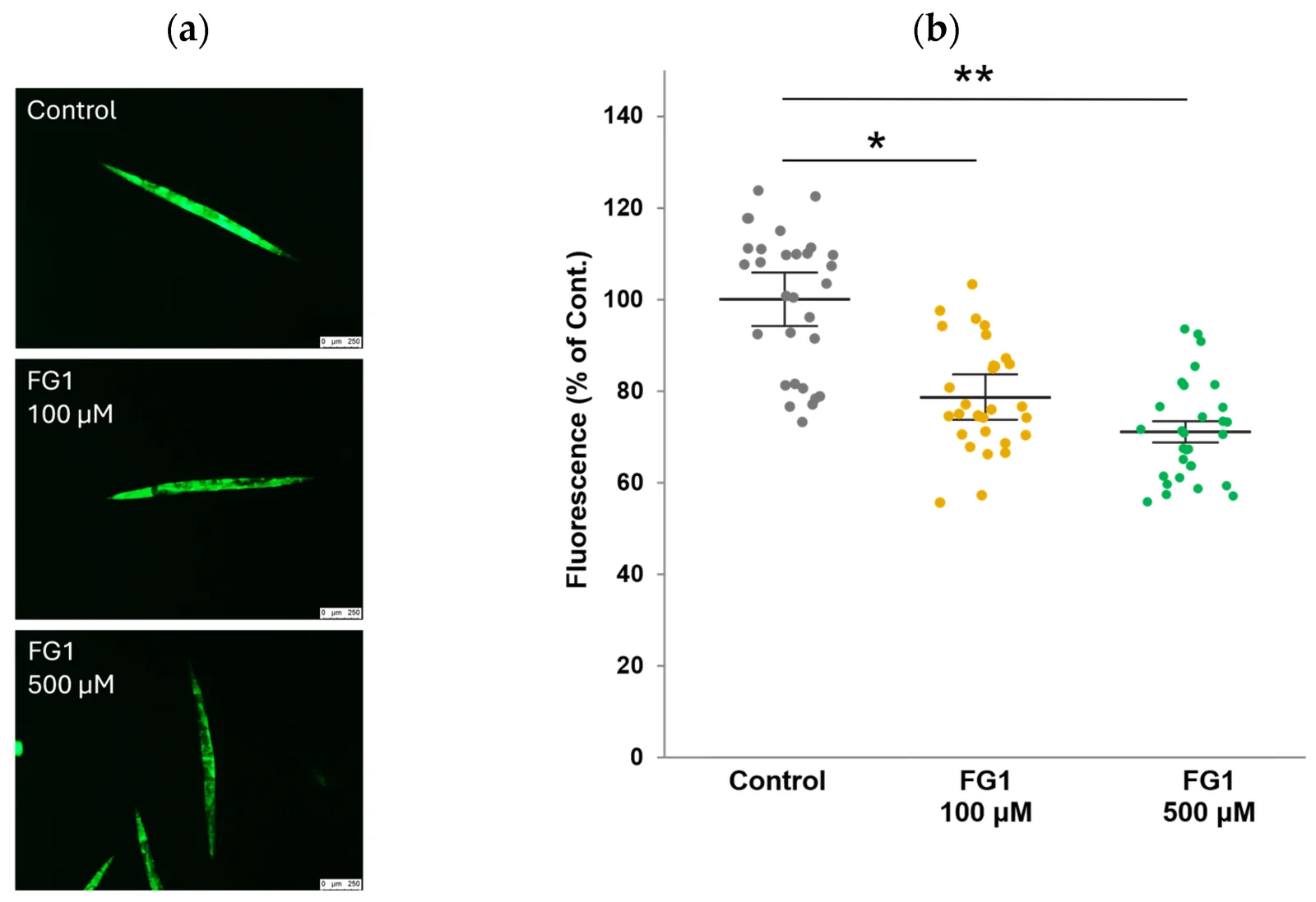
Effects of FG1 on H_2_O_2_ accumulation. Five-day-old N2 worms were cultured on NGM plates with FG1 0, 100 or 500 μM for 10 days (final DMSO concentration: 0.1% in all groups). At 15 days of age, intracellular H_2_O_2_ levels were measured using the fluorescent probe BES-H_2_O_2_-Ac. (**a**) Representative images of worms are shown. (**b**) Fluorescence intensity of BES-H_2_O_2_-Ac was quantified using ImageJ software. Each dot represents an individual worm, and different colours indicate the different FG1 treatment groups. Lines in the scatterplot represent mean ± SD (*n* = 10 worms/trial, 3 independent trials). Asterisks indicate significant differences compared to each control treatment at * *p* < 0.05 and ** *p* < 0.01.

Reactive oxygen species (ROS) levels are associated with age-related phenotypes [12]. To assess endogenous intracellular H_2_O_2_ levels during natural ageing, middle-aged worms cultured on FG1 or control plates were stained with BES-H_2_O_2_-Ac as a fluorescent probe for detecting H_2_O_2_. The intestinal tract and its neighbouring tissues showed significantly stronger staining in the control worms compared to the FG1-treated worms (Figure 6).

These results demonstrate that FG1 effectively suppresses the physiological oxidative damage that occurs during the normal ageing process.

### 2.5. Stress Responses to Exogenous Stimulus

**Figure 7:**
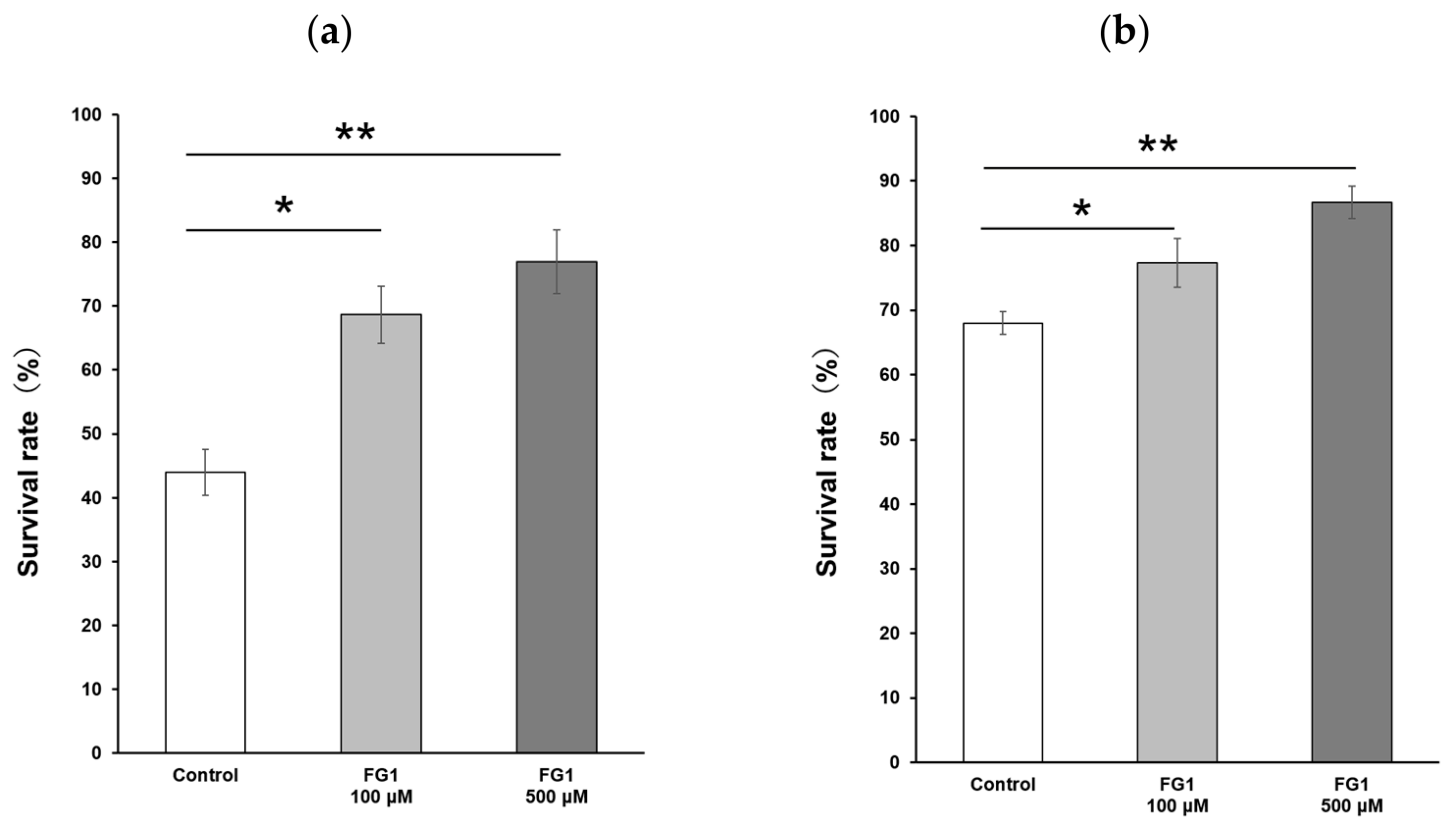
(**a**) Survival rate of heat-stressed worms. Five-day-old N2 worms were cultured on NGM plates with FG1 0, 100 or 500 μM for 5 days (final DMSO concentration: 0.1% in all groups). At 10 days of age, worms were transferred to fresh, treatment-free plates, exposed to 35 °C for 6 h, and allowed to recover at 20 °C for 24 h before survival scoring. Data are expressed as mean ± SD (*n* = 10 worms/trial, 3 independent trials). Asterisks indicate significant differences compared to the control at * *p* < 0.05, ** *p* < 0.01. (**b**) Survival rate of paraquat-stressed worms. Five-day-old N2 worms were cultured on NGM plates with FG1 for 5 days. At 10 days of age, worms were transferred to fresh NGM plates containing 100 mM paraquat and maintained at 20 °C for 24 h before survival scoring. Data are expressed as mean ± SD (*n* = 10 worms/trial, 3 independent trials). Asterisks indicate significant differences compared to the control at * *p* < 0.05, ** *p* < 0.01.

Enhanced stress resistance extends the lifespan of *C. elegans* [13]. Accordingly, we conducted stress resistance assays using various stressors. Heat stress, which induces mitochondrial damage via ROS accumulation [14], was mitigated by FG1, with the highest survival rate observed at 500 µM, indicating a protective effect (Figure 7a). Paraquat, a free radical generator that induces oxidative stress, disrupts cellular structures and impairs nematode lifespan and respiration. Survival of nematodes was significantly higher following FG1 treatment at 100 and 500 µM compared to the untreated controls (Figure 7b). These findings indicate that FG1 enhances resistance to oxidative stress in *C. elegans*.

## 3. Discussion

The free radical theory of ageing proposes that the accumulation of reactive oxygen species (ROS) disrupts redox homeostasis and contributes to oxidative stress and ageing [15,16]. Overproduction of ROS results in oxidative stress, which damages cellular structures, including membranes, lipids, proteins, and DNA, and thus plays a central role in many human diseases and in ageing [17,18,19,20,21,22]. Stress-responsive pathways in *C. elegans* are crucial for lifespan regulation, and enhanced stress resistance is associated with longevity. Consequently, the regulation of oxidative stress has emerged as a key determinant of the lifespan and healthspan of a wide variety of organisms.

In this context, we examined the biological effects of FG1, a butenolide isolated from aromatic vinegar, with a focus on its potential to modulate antioxidant activity and promote longevity in *C. elegans*. Our results demonstrate that FG1 significantly extended the lifespan of *C. elegans* and preserved locomotor activity, suggesting improved physiological resilience during ageing. Moreover, FG1 treatment was associated with reduced H_2_O_2_-related fluorescence and lipofuscin autofluorescence, findings that are consistent with reduced oxidative damage and cellular senescence. In addition, FG1 increased resistance to heat shock and oxidative stress, suggesting that FG1 enhances the capacity of *C. elegans* to cope with multiple forms of cellular stress.

At the molecular level, FG1 treatment upregulated genes encoding detoxification enzymes, including UDP-glycosyltransferases (UGTs) and GSTs. These enzymes are tightly regulated by SKN-1, a transcription factor that functions as the *C. elegans* orthologue of mammalian Nrf proteins. Our observation that FG1 altered the expression of genes known to be associated with SKN-1-regulated detoxification processes is consistent with our previous findings showing FG1-induced Nrf2 activation in mammalian fibroblasts and hepatocytes [23]. Collectively, these results suggest that the biological effects of FG1 are consistent with the involvement of the conserved SKN-1/Nrf2 signalling pathway in enhancing cellular detoxification and antioxidant capacity across species.

The transcription factor SKN-1 is an orthologue of the mammalian Nrf protein, which induces the expression of phase II detoxifying enzymes and antioxidant proteins such as SOD, GST, glutathione peroxidase (GPx), and NAD(P)H:quinone oxidoreductase (NQO-1) [24,25]. This control is mediated by antioxidant response elements (AREs) in the promoters of genes encoding phase II enzymes and antioxidant components.

GSTs and UGTs comprise a large family of enzymes that are generally present in every organism, from bacteria to humans, and are considered major players in phase II detoxification of both endogenous oxidative stress products and exogenous electrophilic chemical compounds [26,27,28]. For example, GST-4 acts by conjugating the reduced form of glutathione (GSH) to a variety of toxic substrates, including damaged lipids and proteins, thereby decreasing their activity and making them more water-soluble, which favours their removal [29,30]. UGTs are the major effectors of phase II metabolism, catalysing conjugative reactions and increasing the solubility and excretion of toxic compounds. The efficacy of the detoxification system is closely associated with ageing and longevity [31]. Oxidative stress derived from exogenous and endogenous substances causes cancer, atherosclerosis, inflammation, hypertension, diabetes, and several age-related diseases [32]. Phase II detoxification enzymes such as GST, glutathione reductase, glutathione peroxidase, UGT, and NAD(P)H:quinone oxidoreductase play major protective roles against oxidants [26,33,34,35]. The activity of these detoxification systems has been associated with cellular protection and longevity. Because oxidative stress contributes to a variety of age-associated pathologies, enhanced activity of endogenous detoxification and antioxidant systems may represent an important mechanism for maintaining cellular homeostasis during ageing.

The use of the DH26 strain in the present study should also be considered when interpreting the transcriptomic findings. DH26 carries a temperature-sensitive mutation in the *rrf-3* gene that causes defective spermatogenesis and complete sterility at 25 °C. The temperature-sensitive sterility of DH26 provides an experimental approach for preventing progeny production without pharmacological sterilization [36]. DH26 has therefore been used in *C. elegans* ageing studies as a sterile strain that avoids potential confounding effects associated with chemical sterilization [37,38]. Importantly, although *rrf-3* is involved in RNA interference sensitivity, genome-wide expression profiling has indicated that, in the absence of exogenous double-stranded RNA, the basal transcriptomic profile of *rrf-3* mutants is highly similar to that of wild-type N2 worms, with relatively limited changes in genes associated with endogenous small interfering RNA pathways [39]. Furthermore, *rrf-3* mutants have been reported to exhibit growth, morphological, and basic physiological characteristics comparable to those of wild-type worms under standard conditions [40]. These findings provide support for the use of DH26 in the present study and suggest that the effects of the *rrf-3* mutation should be taken into consideration but are unlikely to substantially account for the FG1-associated transcriptional changes observed under the experimental conditions used here.

Importantly, FG1 does not exhibit direct radical-scavenging activity, as demonstrated by experiments measuring the consumption of fluorescein in the presence of AAPH (2,2′-azobis(2-amidinopropane) dihydrochloride, a peroxyl radical generator) [23]. The present findings therefore support the possibility that FG1 acts predominantly through indirect antioxidant mechanisms, by enhancing endogenous cellular defence rather than directly neutralizing ROS. The reduction in oxidative stress-associated phenotypes observed in *C. elegans* is consistent with this interpretation. Rather than functioning as a conventional chemical antioxidant, FG1 may enhance cellular homeostasis and stress resistance by modulating endogenous detoxification and stress-response pathways. Such an indirect mechanism could potentially provide broader protection against different types of stress; however, the specific molecular events underlying these effects remain to be determined.

The lifespan-extending effect of FG1 may be associated with the SKN-1 pathway, although the present findings do not establish a definitive requirement for SKN-1. RNA-seq analysis revealed that FG1 treatment modulated not only phase II detoxification genes, such as *ugt* and *gst* family members, but also genes associated with mitochondrial function and oxidative phosphorylation pathways. These transcriptional changes imply that FG1 may influence multiple longevity-related processes in addition to SKN-1-mediated antioxidant defence. Furthermore, KEGG pathway analysis identified enrichment of the longevity-regulating pathway and mitochondrial metabolic pathways, suggesting the possible involvement of broader stress-response and metabolic regulatory networks [3,10,25,41,42,43,44].

Several limitations of the present study should be considered when interpreting these findings. First, although FG1 significantly extended lifespan in wild-type worms, no statistically significant lifespan extension was detected in SKN-1-deficient worms. However, the absence of statistical significance in the mutant strain does not, by itself, demonstrate that the response to FG1 differs significantly between genotypes. A formal genotype × treatment interaction analysis was not performed, and SKN-1 activity was not directly measured. Therefore, the present findings suggest a possible involvement of SKN-1 in the lifespan-extending effect of FG1, but do not establish that SKN-1 is definitively required for this effect. Direct assessment of SKN-1 activity, for example, through nuclear localization analysis or a transcriptional reporter such as *gst-4p*::GFP, together with appropriate statistical comparisons between genotypes, would help clarify the role of SKN-1 in the response to FG1. Second, the RNA-seq findings were not independently validated by RT-qPCR. Thus, the observed changes in detoxification, antioxidant defence, and mitochondrial genes should be interpreted with appropriate caution until independently confirmed. Third, transcriptomic analyses were not performed in SKN-1-deficient worms, and the overlap between FG1-responsive genes and established SKN-1 target genes was not systematically evaluated. Consequently, although the mutant lifespan phenotype and transcriptomic data are consistent with an important role for SKN-1, the extent to which the transcriptional response to FG1 is directly dependent on SKN-1 remains unresolved.

Taken together, our findings indicate that FG1 extends lifespan and enhances stress resistance in *C. elegans* in association with changes in endogenous detoxification, antioxidant defence, and mitochondrial-related pathways. The observation that lifespan extension was statistically significant in wild-type worms but not in SKN-1 mutants is consistent with a possible contribution of SKN-1 to the longevity-promoting effects of FG1; however, the present results do not establish that functional SKN-1 is required for these effects. Importantly, the absence of direct radical-scavenging activity and the changes in endogenous defence-related genes are consistent with a model in which FG1 promotes redox homeostasis primarily through modulation of cellular defence mechanisms rather than direct ROS neutralization. Further studies combining direct measurements of SKN-1 activity, independent validation of transcriptomic changes, and genetic and transcriptomic analyses in *skn-1*-deficient backgrounds will be necessary to define the molecular mechanisms underlying the effects of FG1. Given the evolutionary conservation of the Nrf2-related stress-response system, these findings provide a basis for further investigation of FG1 as a potential modulator of oxidative stress and ageing in higher organisms, including mammals.

## 4. Materials and Methods

### 4.1. Materials

FG1 was chemically synthesized in our laboratory and dissolved in dimethyl sulphoxide (DMSO) before use. The final concentration of DMSO in all treatment solutions, including the control, was 0.1%.

### 4.2. C. elegans Culture Conditions

*C. elegans* N2 Bristol wild-type and mutant strains, including DH26 (*rrf-3(b26)*) and QV225 (*skn-1(zj15)*), were obtained from the Caenorhabditis Genetics Center (St. Paul, MN, USA). Worms were routinely maintained on NGM agar using standard techniques and seeded with OP50, an internationally established feed. Eggs were extracted from egg-bearing worms in a sodium hypochlorite-sodium hydroxide solution and transferred onto NGM plates seeded with OP50. Synchronised L1 worms were grown at 20 °C for the N2 and QV225 strains and at 25 °C for the DH26 strain to obtain L4/young adult worms. The hatching day was defined as day 1; thus, all ages reported in this study refer to days after hatching. For experiments using the fertile N2 and QV225 strains, 5-fluoro-2′-deoxyuridine (FUDR) was added at the L4 stage to prevent progeny production. For the DH26 strain, progeny production was minimized by maintaining the worms at 25 °C throughout development and adulthood.

FG1 treatment was initiated when the nematodes reached the 5-day-old adult stage. This timing was selected to evaluate the compound’s effects specifically on the ageing process rather than on larval development. The 5-day-old adult stage represents an early adulthood stage in which development is complete and age-related physiological deterioration is minimal, allowing for clear observation of age-associated functional changes induced by FG1 exposure.

### 4.3. C. elegans Lifespan Assay

For the lifespan assay, DH26 mutants were used because they are unable to produce progeny at 25 °C without altering the *C. elegans* phenotype [36]. Synchronised L1 larvae were fed OP50 and maintained until they reached the 5-day-old stage. The resultant synchronised hermaphrodites were transferred to NGM plates containing 0, 100, and 500 μM of FG1 (12 worms per 35 mm plate). FG1 stock solution was dissolved in DMSO, and all test plates, including the 0 μM control group, contained a matching final concentration of 0.1% (*v*/*v*) DMSO as the vehicle control. After the plates were incubated, the number of live or dead worms was counted every 2 days. Lifespan was measured from the day of hatching, with the day of hatching defined as day 1. Worms were inferred as “dead” when they failed to respond to a gentle touch with a worm picker. Worms that crawled off the plate and underwent non-natural death, such as internal hatching or adherence to the plate wall, were considered lost and excluded from the analysis. More than 65 worms from each group were used in the longevity assays. The experimental procedures were repeated three times to ensure the robustness and reliability of the results.

### 4.4. Locomotion Assay

Five-day-old N2 Bristol wild-type worms were placed on NGM plates seeded with varying concentrations of FG1 and maintained at 20 °C until they were 10 days old. Locomotor performance was assessed by measuring the body bending rate. Live 10-day-old worms were placed in S-basal buffer, allowed to recover for 2 min, and the number of body bends was counted for 1 min. Each experimental condition was evaluated across three independent trials, using 10 worms per trial.

### 4.5. RNA Sequencing and Data Analysis

#### 4.5.1. Sample Preparation

Approximately 2000 worms per sample were collected for RNA-seq. Five-day-old DH26 mutant worms were transferred to NGM plates with or without 500 μM FG1 and allowed to develop into 6-day-old worms. The 100 μM FG1 condition was not included in the transcriptomic analysis, as 500 μM produced the most prominent physiological effects in phenotypic assays. Worms were collected and washed three times with S-basal buffer. Three independent biological replicates (*n* = 3 per group) were prepared for both the untreated Control (0 μM) and FG1-treated (500 μM) groups.

#### 4.5.2. mRNA Sequencing

Total RNA was extracted with TRIzol Reagent (Eugene, OR, USA) following the manufacturer’s protocol and stored at −20 °C. RNA quality was evaluated using a NanoPhotometer spectrophotometer (IMPLEN, Munich, Germany), and RNA integrity was characterised using a Bioanalyzer 2100 (Agilent, Santa Clara, CA, USA). All RNAs were confirmed to be intact. Libraries for mRNA sequencing were created using the NEBNext Ultra II RNA Library Prep Kit for Illumina and the NEBNext Poly(A) mRNA Magnetic Isolation Module (New England Biolabs, Ipswich, MA, USA) following the manufacturer’s instructions. The concentration and size distribution of the libraries were measured using the Agilent DNA 7500 kit (Agilent) on a Bioanalyzer. All samples were analysed using NGS. The libraries were pooled, and the concentrations were adjusted to 10 nM. The pooled libraries were denatured and neutralised by further dilution. Subsequently, the libraries were subjected to NGS on a NovaSeq X Plus System (Illumina, San Diego, CA, USA) using the 10 B Reagent Kit (300 cycles) (Illumina). Sequencing was performed using paired-end reads of 150 bases. After the sequencing run, FASTQ files were exported, and basic information of the NGS run data was checked for quality control using CLC Genomics Workbench software (version 24.0; CLC, Qiagen, Venlo, The Netherlands). The results showed that 99.83% of all reads had a Phred score > 20, confirming successful data acquisition.

#### 4.5.3. Bioinformatic Analysis

Bioinformatic analysis was performed using CLC Genomics Workbench (version 24.0; CLC, Qiagen, Venlo, The Netherlands). FASTQ reads were mapped to the *C. elegans* reference sequence (WBcel235). Statistical differential expression analysis was performed using the ‘Empirical analysis of DGE’ tool in CLC Genomics Workbench, which implements the Exact Test algorithm based on the edgeR package (version 3.4.0) under the assumption of a Negative Binomial distribution for read counts. *p*-values were adjusted for multiple testing using the Benjamini–Hochberg False Discovery Rate (FDR) method. Genes meeting the cutoff criteria of FDR-adjusted *p*-value < 0.05 and |fold change| ≥ 1.5 were identified as differentially expressed genes (DEGs). Gene enrichment and Gene Ontology (GO)/KEGG pathway analyses were performed using Metascape [45] based on the filtered DEG list.

### 4.6. Thermorecovery Assay

To assess thermal shock resistance, 5-day-old N2 Bristol wild-type worms were placed on NGM plates seeded with varying concentrations of FG1 until they were 10 days old, and then transferred to new treatment-free NGM plates and incubated at 35 °C for 6 h. Thereafter, the worms were allowed to recover at 20 °C for an additional 24 h, and the numbers of dead and live worms were determined. Each experimental condition was evaluated across three independent trials, using 10 worms per trial.

### 4.7. Stress Responses for Paraquat

To assess resistance to paraquat-induced stress, 5-day-old N2 Bristol wild-type worms were placed on NGM plates seeded with varying concentrations of FG1 and maintained until they reached at 10 days of age. The worms were then transferred to new NGM plates seeded with a final concentration of 100 mM paraquat. Thereafter, the worms were maintained at 20 °C for an additional 24 h, after which the numbers of live and dead worms were determined. Each experimental condition was evaluated across three independent trials, using 10 worms per trial.

### 4.8. Lipofuscin Accumulation in Living Worms

Five-day-old N2 Bristol wild-type worms were placed on NGM plates seeded with varying concentrations of FG1 until they were 15 days old. Randomly selected worms were washed with S-basal buffer for 30 min and placed on 3% agar pads coated with 1 M sodium azide for anaesthesia. Lipofuscin autofluorescence images were obtained by excitation at 340–380 nm and emission at 425 nm using a LEICA imaging system (DM4 B; Leica Microsystems, Wetzlar, Germany). Lipofuscin-positive areas were quantified by densitometry with ImageJ software (version 1.54p, National Institutes of Health, Bethesda, MD, USA). Each experimental condition was evaluated across three independent trials, using 10 worms per trial.

### 4.9. Fluorescent Staining of H_2_O_2_ in Living Worms

Intracellular H_2_O_2_ levels were assessed in *C. elegans* using the fluorescent probe BES-H_2_O_2_-Ac (Fujifilm Wako Pure Chemical, Tokyo, Japan). The probe was diluted to 200 μM in S-basal buffer. Synchronised 5-day-old N2 Bristol wild-type worms were treated with varying concentrations of FG1 on NGM plates until they were 15 days old. Ten worms per condition were incubated in 450 μL of staining solution for 1 h, washed with S-basal buffer (30 min), and anaesthetised with 1 M sodium azide on 3% agar pads. Fluorescence was detected using a LEICA DM4 B microscope (excitation: 460–500 nm; emission: 512–542 nm). Each experimental condition was evaluated across three independent trials, using 10 worms per trial.

### 4.10. Statistical Analysis

For the lifespan assays, survival curves were analyzed separately for each of three independent experimental trials using the log-rank test. For the locomotion, lipofuscin accumulation, H_2_O_2_ accumulation, and stress resistance assays, 10 worms were examined per treatment group in each of three independent experimental trials. For each trial, measurements from the 10 worms were summarized as a single value for each treatment group. Statistical analyses were performed using these trial-level values, with each independent trial treated as the experimental unit (*n* = 3 per group). Differences among groups were analyzed using one-way ANOVA followed by Bonferroni’s post hoc test.

Statistical analyses were conducted using BellCurve for Excel (SSRI, Tokyo, Japan). All data are expressed as mean ± SD. Differences were considered statistically significant at *p* < 0.05.

## Figures and Tables

**Table 1:** Effects of FG1 on mean, median and maximum lifespan of DH26 strains. (from 3 replicates).

Strain	Group	Mean Lifespan (Days)	Median Lifespan (Days)	Max. Lifespan (Days)	Number of Nematodes
DH26	Control	18.5 ± 0.2	19.7 ± 1.2	25.3 ± 1.2	240/252
DH26	FG1 100 µM	19.5 ± 0.3	22.0 ± 0.0	28.7 ± 1.2	234/252
DH26	FG1 500 µM	20.7 ± 0.1	24.3 ± 1.2	32.0 ± 2.0	231/252

Data are expressed as mean ± SD, *n* = 3.

**Table 2:** Effects of FG1 on mean, median and maximum lifespan of N2 and QV225 strains. (from 3 replicates).

Strain	Group	Mean Lifespan (Days)	Median Lifespan (Days)	Max. Lifespan (Days)	Number of Nematodes
N2	Control	24.3 ± 1.1	26.0 ± 0.0	32.7 ± 1.2	210/252
N2	FG1 500 µM	28.7 ± 1.2	32.7 ± 1.2	42.0 ± 2.0	201/252
QV225	Control	23.3 ± 1.1	24.7 ± 1.2	31.3 ± 1.2	230/252
QV225	FG1 500 µM	24.8 ± 1.1	24.0 ± 0.0	32.0 ± 0.0	232/252

Data are expressed as mean ± SD, *n* = 3.

## Data Availability

The experimental data generated in this study are available from the corresponding author upon reasonable request. The RNA-Seq data analyzed in this study have been deposited in the NCBI Gene Expression Omnibus (GEO) database and are accessible through GEO Series accession number GSE327594. Further inquiries can be directed to the corresponding author.
